# A large outbreak of invasive Group B *Streptococcus* Sequence Type 283 infection linked to physical contact of freshwater fish

**DOI:** 10.1017/S0950268825100186

**Published:** 2025-06-24

**Authors:** Hoi-kei Wong, Kai-lok Lam, Alan Ka-lun Tsang, Derek Ling-lung Hung, Tonny Ng, Albert Ka-wing Au, Edwin Lok-kin Tsui

**Affiliations:** 1 Communicable Disease Branch, Centre for Health Protection, Department of Health, Hong Kong Special Administrative Region, China; 2 Public Health Laboratory Services Branch, Centre for Health Protection, Department of Health, Hong Kong Special Administrative Region, China; 3 Controller of the Centre for Health Protection (CHP), Department of Health, Hong Kong Special Administrative Region, China

**Keywords:** freshwater fish, Group B *Streptococcus*, physical contact, Sequence Type 283

## Abstract

Invasive Group B *Streptococcus* (GBS) infection caused by the highly virulent Sequence Type 283 (ST283) strain has been linked to consumption of raw freshwater fish. In late summer 2024, enhanced surveillance in Hong Kong detected a surge of invasive ST283 cases.

A retrospective case–control study was conducted involving all invasive GBS patients reported during August to September 2024. Data were collected through standardised interviewer-administered questionnaires. Cases were defined as patients infected with the ST283 strain, while controls had non-ST283 strains. A multivariate logistic regression analysis was conducted to determine the risk factors.

Among 170 invasive GBS patients, 131 (77%) were identified as cases and 39 (23%) as controls. Physical handling of raw freshwater fish was found to be the strongest risk factor for ST283 infection (adjusted odds ratio: 8.4, 95% confidence interval: 1.4–50.1).

This study represents the first epidemiological evidence specifically linking physical contact with raw freshwater fish to an increased risk of invasive GBS ST283 infection. Effective interdepartmental coordination, intensive public health education, active surveillance, and prompt environmental interventions effectively mitigated this large outbreak. The findings underscore the need for sustainable preventive strategies targeting high-risk fish handling practices, particularly during warm periods favouring environmental proliferation of ST283.

## Background

Group B *Streptococcus* (GBS), or *Streptococcus agalactiae*, is a Gram-positive, non-motile, encapsulated coccus bacterium. It is found in a variety of species, including humans, mammals, amphibians, reptiles, and fish. In humans, GBS colonises the gastrointestinal and genitourinary tracts as a commensal organism in 20 to 40% of healthy adults. Since the 1960s, GBS has been increasingly recognised as a common pathogen of severe invasive diseases among vulnerable populations [1].

GBS expresses a polysaccharide capsule with 10 distinct serotypes. ST283 belongs to Subtype 4 of Serotype III, wherein within the subtype, the strains are indistinguishable by pulse-field gel electrophoresis, possessing characteristic C-α protein and C-α-repetitive sequences and IS1381 insertion sequence [2].

Compared with other GBS strains, ST283 is associated with higher mortality and invasive diseases, in particular among otherwise healthy adults and adults with relatively few underlying comorbidities. ST283 has been detected in freshwater fish in Southeast Asian countries, with prevalence varying between 12.5 and 100% [3].

In 2015, Singapore experienced a major foodborne outbreak involving more than 100 cases of invasive ST283 infection. Epidemiological investigation indicated that the outbreak had a strong association with the consumption of raw freshwater fish, including raw Asian bighead carp and snakehead [4,5,6]. Following this, cases of invasive GBS ST283 have continuously been reported in and around Southeast Asia [7,8]. While raw freshwater fish consumption is the sole well-established risk factor to date [9], consolidated data do not exist on other potential risk factors [1]. Given ST283’s high virulence and emerging zoonotic potential, it is crucial to better understand the whole spectrum of associated factors. This knowledge will enable the development of more targeted preventive and control strategies, including precise health education messages.

In September 2021, Hong Kong experienced a rise in invasive GBS cases attributed to the ST283 strain. Epidemiological investigation conducted by the Centre for Health Protection (CHP) suggested handling raw freshwater fish and consuming undercooked freshwater fish as possible risk factors [10]. However, there remained a paucity of direct evidence and data in the scientific literature specifically examining the role of physical contact with contaminated freshwater fish versus consumption of undercooked freshwater fish in the transmission of ST283.

In late August 2024, again during the hot summer season, enhanced laboratory-based surveillance on invasive GBS cases conducted by CHP and the Hospital Authority (HA), the statutory body managing all public hospitals in Hong Kong (accounting for more than 90% of inpatient admissions territory-wide), detected an upward trend in the number of patients tested positive for GBS from normally sterile body sites, including blood, joint fluid, and cerebrospinal fluid. Whole-genome sequencing confirmed that this increase was attributable to the ST283 strain of GBS.

## Methodology

CHP conducted a retrospective case–control study as part of the territory-wide outbreak investigation. All invasive GBS cases reported by the HA managing 43 public hospitals and institutions during the outbreak period were investigated and included in this retrospective study. The outbreak period is defined as the timeframe during which the weekly number of in-patients with invasive GBS infection exceeds the average of the past 10 years by 2 standard deviations. In this analysis, the period spans from late August to September 2024.

We conducted telephone interviews with all those with GBS detected in blood, joint fluid, or cerebrospinal fluid using a standardised questionnaire administered by trained nurses. Upon informed consent, the information was obtained from the patients (or their informants for those cases that were incapacitated by illness) as soon as the invasive GBS cases were reported. Socio-demographic data, medical history, travel history, and 1-month exposure histories (including fish and other exposures) were obtained. For fish exposure, we conducted a detailed inquiry into the handling practices, covering aspects such as where the fish was purchased, the processes involved, washing or rinsing, rubbing, descaling, chopping, cooking steps, and whether gloves were used. Of note, in Hong Kong, fresh food products are often purchased from traditional wet markets that also sell fresh meat and fish [11].

Upon the availability of whole-genome sequencing-based multi-locus sequence typing results performed by CHP’s Public Health Laboratory Services Branch, the subjects were categorised as either ST283 (cases) or non-ST283 (controls). We also carried out environmental sampling at four of the wet retail markets patronised by the ST283 cases, and a wholesaler and three local fish farms. Statistical analysis is conducted by logistic regression performed via IBM SPSS Statistics Version 30.0.0.0 (172). A two-sided *p* < 0.05 is considered statistically significant.

## Results

Out of the total of 170 invasive GBS patients, sequence typing identified 131 cases (belonging to the ST283 strain) and 39 controls (belonging to non-ST283 strains). Characteristics of the cases and controls are summarised in Table 1. Out of the 131 ST283 cases, 61 (46.6%) were males and 70 (53.4%) females, with ages spanning from 29 to 97 years (median: 69). Overall, the ST283 cases tended to be younger, with a median age of 69 years compared to 74 years among controls. A slightly larger proportion was female (53.4% vs. 46.2%). Among the ST283 cases, 76% reported having any underlying medical conditions, which is less than that reported for the controls (92%).

**Table 1.** Characteristics of cases and controls, and results of univariate and multivariate analyses

		Case (*n* = 131)	Control (*n* = 39)	Univariate analysis	Multivariate analysis
Sex	Female Male	70 (53%) 61 (47%)	18 (46%) 21 (54%)	Not significant	Not significant
Years of age	Range Median	29–97 69	0–95 74	Not significant	Not significant
No comorbidities	Yes No	31 (24%) 100 (76%)	3 (8%) 36 (92%)	*p* = 0.039 Odds ratio: 3.7 (1.1–12.9)	*p* = 0.045 Adjusted odds ratio: **5.2** (1.0–25.8)
Pre-existing wound	Yes No	17 (13%) 114 (87%)	7 (18%) 32 (82%)	Not significant	Not significant
Pre-existing antibiotic intake	Yes No	4 (3%) 127 (97%)	2 (5%) 37 (95%)	Not significant	Not significant
Daily work involving handling of freshwater fish (including housewife)	Yes No	30 (23%) 101 (77%)	5 (13%) 34 (87%)	Not significant	Not significant
Residential district	HK Island Kowloon NT	21 (16%) 52 (40%) 58 (44%)	8 (21%) 12 (31%) 19 (49%)	Not significant	Not significant
Recent travel outside Hong Kong	Yes No	31 (24%) 100 (76%)	3 (8%) 36 (92%)	*p* = 0.039 Odds ratio: 3.7 (1.1–12.9)	Not significant
**Physical contact related**					
Handled^a^ freshwater fish	Yes No	74 (56%) 57 (44%)	5 (13%) 34 (87%)	*p* = <0.001 Odds ratio: 8.8 (3.2–24.0)	*p* = 0.019 Adjusted odds ratio: **8.4** (1.4–50.1)
Handled^a^ with bare hands	Yes No	45 (34%) 86 (66%)	3 (8%) 36 (92%)	*p* = 0.003 Odds ratio: 6.3 (1.8–21.5)	Not significant
Injury during handling^a^	Yes No	10 (8%) 121 (92%)	1 (3%) 38 (97%)	Not significant	Not significant
Contact during purchase^b^	Yes No	99 (76%) 32 (24%)	13 (33%) 26 (67%)	*p* = <0.001 Odds ratio: 6.2 (2.8–13.4)	*p* = 0.043 Adjusted odds ratio: **2.8** (1.0–7.8)
Injury during purchase^b^	Yes No	13 (10%) 118 (90%)	1 (3%) 38 (97%)	Not significant	Not significant
**Consumption related**					
Consumed freshwater fish	Yes No	88 (67%) 43 (33%)	19 (49%) 20 (51%)	*p* = 0.038 Odds ratio: 2.2 (1.0–4.5)	Not significant
Consumed undercooked freshwater fish	Yes No	4 (3%) 127 (97%)	1 (3%) 38 (97%)	Not significant	Not significant
Consumed sashimi	Yes No	22 (17%) 109 (83%)	3 (8%) 36 (92%)	Not significant	Not significant
Often eat (not necessarily freshwater fish) outside home	Yes No	32 (24%) 99 (76%)	9 (23%) 30 (77%)	Not significant	Not significant

a This refers to direct handling or manipulation of freshwater fish for food preparation.

b This refers to the physical contact of freshwater fish during purchase.

**Figure 1.**
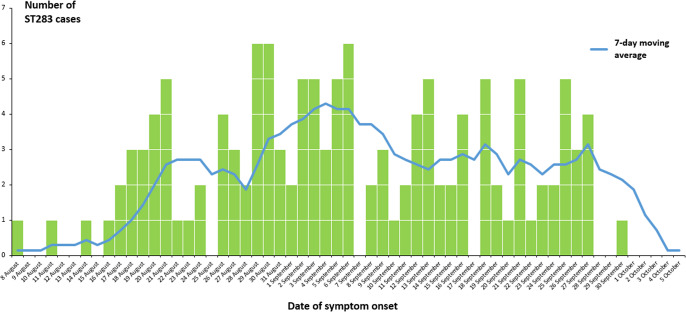
Epidemic curve of the ST283 cases.

The ST283 patients comprised both retirees and people from a range of occupations. Their onset dates ranged from 8 August to 30 September 2024 (Figure 1). The clinical presentations primarily involved sepsis (68; 52%), joint abscesses (39; 30%), and meningitis (10; 8%). Among them, four (3%) succumbed to the invasive GBS ST283 infection.

Univariate logistic regression analysis found that several exposure variables were significantly associated with increased odds of belonging to ST283 cases relative to non-ST283 controls (Table 1). These included having no documented comorbidities, recent travel outside Hong Kong, physical contact with freshwater fish during purchase, physical contact with freshwater fish through direct manual handling during food preparation, using bare hands during the direct manual handling, and consuming freshwater fish. In contrast, we found no statistically significant association for demographic factors like age, sex and, district of residence.

A multivariate model controlling for all relevant covariates showed that direct manual handling of raw freshwater fish during food preparation remained the strongest independent risk factor, with an adjusted odds ratio of 8.4 (95% confidence interval: 1.4–50.1) for ST283 infection compared to non-handlers after adjusting for other variables. Other physical contact (e.g., during purchasing) also remained a significant risk factor (adjusted odds ratio: 2.8, 95% confidence interval: 1.0–7.8).

The freshwater fish involved included a variety of species, with grass carp being the most common. These fish were purchased from a range of more than 50 markets throughout all districts. There were no common food premises patronised by the cases either.

Two freshwater fish samples and three environmental swabs obtained from a freshwater retail stall in a wet market tested positive for ST283, while the other 97 freshwater fish samples and environmental swabs taken tested negative for ST283. Other sequence types were not analysed. Genome sequencing revealed that the ST283-positive samples matched those from the human cases.

## Discussion

The results from this case–control study and multivariate logistic regression analyses clearly implicate direct manual handling of raw freshwater fish as the key risk factor for this outbreak, with an adjusted odds ratio of 8.4 (95% confidence interval: 1.4–50.1) after controlling for potential confounding variables.

This association may be attributable to the ability of the bacteria to penetrate micro-abrasions or cuts, as the skin can have tiny cuts or abrasions that are not visibly apparent. Indeed, handling rough surfaces, including fish scales or fins, can create these micro-injuries, particularly when handling is performed with bare hands. In addition, the presence of moisture during fish handling may further soften the skin barrier and make it more susceptible to penetration by bacteria when the fish is contaminated [12]. This transmission route is consistent with observations that none of the cases involved professional fish handlers, such as fishmongers or individuals engaged in fish culture-related jobs, who are always equipped with proper protective equipment like gloves and possess relevant knowledge and skills.

While handling raw freshwater fish with bare hands was identified as a risk factor in univariate analysis, it did not remain statistically significant after multivariate adjustment. This suggests that the initial association may be more accurately attributed to underlying handling behaviours, such as inconsistent or improper use of gloves, rather than to bare-hand handling itself.

In contrast, our findings showed that simply consuming any undercooked freshwater fish is not identified as a significant risk factor after controlling for other variables like handling in the multivariate model. This may be attributed to our extensive and long-standing publicity efforts aimed at raising awareness about the dangers of consuming raw or undercooked fish. As a result, relatively few individuals (four cases and one control only) reported consuming undercooked freshwater fish, which could limit the statistical power to detect any potential association.

The absence of geographical clustering of cases by residential district and the wide distribution of retail wet markets where implicated fish were purchased ruled out a single common source for this outbreak. While the precise origins or original introduction of this ST283 strain remains unknown, the detection of isolates with almost identical whole-genome sequences from two freshwater fish samples and three environmental swabs collected from a freshwater fish stall in a retail market reinforces the epidemiological link with raw fish handling.

The findings of this study, particularly the relatively young age and low comorbidity burden among ST283 cases, are consistent with previous literature reporting the increased virulence of this strain in causing invasive diseases among otherwise healthy individuals with relatively few underlying comorbidities [4,5,6].

In response to the outbreak, relevant government departments promptly conducted cleansing and thorough disinfection in the relevant markets and inspected all fish stalls, licenced fresh provision shops, and permitted premises selling freshwater fish in Hong Kong. They also offered hygiene education to relevant operators, advising them to perform deep cleaning and disinfection after business hours. The CHP enhanced territory-wide publicity and health education to increase public awareness of invasive GBS ST283 infection through various channels, including press releases, social media, and media interviews. It also advised the public to consult a doctor immediately if they experienced relevant symptoms, alerted medical practitioners to stay vigilant against invasive GBS ST283 infection, and collaborated with the HA to monitor new cases daily to identify any possible cases as far as possible.

With these swift and effective measures and the collaborative efforts of the Government, the trend of invasive GBS cases has gradually declined since the peak in early September 2024. The number of invasive GBS cases has largely reverted to the level seen in early August before the outbreak occurred.

The strength of this study lies in its minimal recall and interview bias, as the exposure history was collected soon after symptom onset and before the sequence typing results were available. In contrast, the weakness is the relatively small number of non-ST283 controls available for analysis, which could limit the statistical power of the study.

In conclusion, this outbreak of ST283 was strongly linked to the direct handling of raw freshwater fish, as indicated by the epidemiological findings and statistical analyses. This study is the first of its kind showing this association, which is vital in formulating targeted health advice. The outbreak also highlighted the importance of effective interdepartmental coordination in implementing various measures to control the outbreak. The existing surveillance system promptly detected the increase in cases, leading to an immediate epidemiological investigation to determine associated factors. The intensive publicity and public education efforts conducted in September 2024 effectively altered the behaviour of the general public, resulting in a rapid decrease in exposure and a decline in case numbers.

The global literature on GBS ST283 remains relatively limited, with significant data gaps along the whole freshwater fish supply chain and, as a result, the risk posed by GBS ST283 from freshwater fish remains highly uncertain [1]. A local [13] and some overseas [1] studies suggest that environmental factors, such as higher temperatures, may influence the proliferation of GBS ST283. GBS isolates from freshwater fish have shown optimal growth at 30 °C, and invasive GBS ST283 cases are mainly reported from Southeast Asia, which is a region with a hot climate. Considering the local outbreaks in Hong Kong during the summer months of 2021 and 2024, it is plausible that similar increases in GBS ST283 activity linked to freshwater fish could occur again in future summers.

Therefore, it is imperative to enhance interdepartmental collaboration to raise public awareness about related preventive measures. This includes not only refraining from eating raw or undercooked freshwater fish but also wearing appropriate protective gear and practicing good hand hygiene when making physical contact with freshwater fish. It is essential to enhance surveillance efforts to identify any abnormal or unusual signals before the summer season. This proactive approach will facilitate the timely implementation of targeted preventive measures based on the effective practices used during the recent outbreak response.

## Data Availability

Data are provided within the manuscript.

## Declarations

The authors assert that all procedures contributing to this work comply with the ethical standards of the relevant national and institutional committees on human experimentation and with the Helsinki Declaration of 1975, as revised in 2008.
